# Regional spinal cord volumes and pain profiles in AQP4-IgG + NMOSD and MOGAD

**DOI:** 10.3389/fneur.2024.1308498

**Published:** 2024-01-26

**Authors:** Susanna Asseyer, Ofir Zmira, Laura Busse, Barak Pflantzer, Patrick Schindler, Tanja Schmitz-Hübsch, Friedemann Paul, Claudia Chien

**Affiliations:** ^1^Charité–Universitätsmedizin Berlin, corporate member of Freie Universität Berlin-Universität zu Berlin and Max Delbrück Center for Molecular Medicine in the Helmholtz Association, Experimental and Clinical Research Center, Humboldt, Germany; ^2^Charité–Universitätsmedizin Berlin, corporate member of Freie Universität Berlin-Universität zu Berlin, Neuroscience Clinical Research Center, Humboldt, Germany; ^3^Department of Neurology, Charité–Universitätsmedizin Berlin, corporate member of Freie Universität Berlin and Humboldt-Universität zu Berlin, Berlin, Germany; ^4^Department of Neurology, Sheba Medical Center, Ramat Gan, Tel Hashomer, Israel; ^5^Department of Neurology and Neurosurgery, Sackler Faculty of Medicine, Tel Aviv University, Tel Aviv, Israel; ^6^Department for Psychiatry and Psychotherapy, Charité–Universitätsmedizin Berlin, corporate member of Freie Universität Berlin, Humboldt-Universität zu Berlin, Berlin, Germany

**Keywords:** AQP4-IgG, MOG-IgG, NMOSD, MOGAD, spinal cord, pain, MRI

## Abstract

**Objective:**

Aquaporin-4-antibody-seropositive (AQP4-IgG+) Neuromyelitis Optica Spectrum Disorder (NMOSD) and Myelin Oligodendrocyte Glycoprotein Antibody-Associated Disorder (MOGAD) are relapsing neuroinflammatory diseases, frequently leading to chronic pain. In both diseases, the spinal cord (SC) is often affected by myelitis attacks. We hypothesized that regional SC volumes differ between AQP4-IgG + NMOSD and MOGAD and that pain intensity is associated with lower SC volumes. To evaluate changes in the SC white matter (WM), gray matter (GM), and pain intensity in patients with recent relapses (myelitis or optic neuritis), we further profiled phenotypes in a case series with longitudinal imaging and clinical data.

**Methods:**

Cross-sectional data from 36 participants were analyzed in this retrospective study, including 20 AQP4-IgG + NMOSD and 16 MOGAD patients. Pain assessment was performed in all patients by the Brief Pain Inventory and painDETECT questionnaires. Segmentation of SC WM, GM, cervical cord volumes (combined volume of WM + GM) was performed at the C2/C3 cervical level. WM% and GM% were calculated using the cervical cord volume as a whole per patient. The presence of pain, pain severity, and clinical disability was evaluated and tested for associations with SC segmentations. Additionally, longitudinal data were deeply profiled in a case series of four patients with attacks between two MRI visits within one year.

**Results:**

In AQP4-IgG + NMOSD, cervical cord volume was associated with mean pain severity within 24 h (β = −0.62, *p* = 0.009) and with daily life pain interference (β = −0.56, *p* = 0.010). Cross-sectional analysis showed no statistically significant SC volume differences between AQP4-IgG + NMOSD and MOGAD. However, in AQP4-IgG + NMOSD, SC WM% tended to be lower with increasing time from the last attack (β = −0.41, *p* = 0.096). This tendency was not observed in MOGAD. Our case series including two AQP4-IgG + NMOSD patients revealed SC GM% increased by roughly 2% with either a myelitis or optic neuritis attack between visits. Meanwhile, GM% decreased by 1–2% in two MOGAD patients with a myelitis attack between MRI visits.

**Conclusion:**

In AQP4-IgG + NMOSD, lower cervical cord volume was associated with increased pain. Furthermore, cord GM changes were detected between MRI visits in patients with disease-related attacks in both groups. Regional SC MRI measures are pertinent for monitoring disease-related cord pathology in AQP4-IgG + NMOSD and MOGAD.

## 1 Introduction

Neuromyelitis optica spectrum disorders (NMOSD) are severe and relapsing inflammatory diseases of the central nervous system (CNS). Attacks occur mainly in the spinal cord (SC) and optic nerves and lead to persistent damage; however, diencephalon and cerebral attacks are also well described (1, 2). Pathogenic serum IgG antibodies against aquaporin-4 (AQP4-IgG) can be detected in ~70% of NMOSD patients (3).

Myelin oligodendrocyte glycoprotein (MOG) antibody-associated disorder (MOGAD) is a different disease entity that also affects the CNS with symptoms of optic neuritis, myelitis, and/or encephalomyelitis (4).

MOGAD and AQP4-IgG-seropositive (AQP4-IgG+) NMOSD have distinct pathogenesis. While AQP4-IgG + NMOSD causes complement activation resulting in astrocytic death, pathological specimens from MOGAD are more similar to demyelination in MS, with T-cells and macrophages found around blood vessels and with relative preservation of oligodendrocytes (5, 6).

Spinal cord affection by myelitis, in the form of longitudinally extensive transverse myelitis (LETM) or shorter lesions, occurs in both diseases (5). Chronic LETM/myelon lesions can cause SC atrophy and have been found to be significantly more prevalent in AQP4-IgG + NMOSD than in MOGAD (7). Meanwhile, multiple locations (≥2) of SC and SC gray matter (GM) hyperintense lesions (H-sign) are presented more often in MOGAD patients (8). AQP4-IgG + NMOSD patients have been shown to have reduced GM cord volumes localized to lesion regions, whereas MOGAD patients had reduced GM cord volumes only when lesions were not resolved (9).

Clinically, patients with LETM typically present with paresis, sensory deficits, bowel, and bladder disturbances, and intense neuropathic pain (10–13). However, myelitis is only one of many triggers causing pain in NMOSD and MOGAD. Pain syndromes in NMOSD and MOGAD comprise headache, neuropathic pain, and musculoskeletal pain, including spasticity, painful tonic spasms, and back pain (14). Especially, in AQP4-IgG + NMOSD, pain is frequently chronic over the disease course and is one of the most disabling symptoms in over 80% of the patients (13, 15). Chronic pain is less prevalent in MOGAD (14), which may be linked to better recovery of acute lesions. Currently, it is unclear if non-myelitis attacks affect the SC or if myelitis attacks affect other CNS regions (16, 17), and also if subclinical affection relates to the pain experienced by patients.

This study aims to investigate the relationship between cervical SC volumes as well as white matter (WM) percentages and pain intensity in AQP4-IgG+NMOSD and MOGAD.

We hypothesized that different SC regional measures, in particular, WM percentages, between MOGAD and AQP4-IgG + NMOSD, will be observed.

We further aimed to profile in detail, SC magnetic resonance imaging (MRI), pain, and clinical data collected from subjects with relapses in between two consecutive visits as a case series in the two disease groups.

## 2 Methods

### 2.1 Participants

Cross-sectional data from 51 participants from an ongoing observational study in NMOSD and MOGAD (Experimental and Clinical Research Center, Charite—Universitätsmedizin Berlin) were screened for inclusion. Inclusion criteria were a minimum age of 18 years and seropositivity for antibodies against AQP4 in a fixed cell-based assay or MOG in a live cell-based assay at any time during the disease, along with the availability of SC MRI. Patients who were seronegative for AQP4-IgG and MOG-IgG or did not receive SC MRI were excluded. A total of 39 patients fulfilled the inclusion criteria. Twenty-three patients had a diagnosis of AQP4-IgG + NMOSD according to the international consensus diagnostic criteria for NMOSD 2015 (18). Due to MRI quality issues (motion artifacts), four AQP4-IgG + NMOSD participants were removed from the cohort. Fifteen subjects had a diagnosis of MOGAD according to the new MOGAD criteria (4). One subject with low-positive MOG-IgG titer (AQP4-IgG negative) and recurrent ON did not fulfill the MOGAD criteria by Banwell et al. but was included in the study as part of the MOGAD group. In our cross-sectional analysis, 20 AQP4-IgG + NMOSD and 16 MOGAD patients with previous disease-related attacks were included. Previous attacks consisted of myelitis, optic neuritis, brainstem attacks, and cerebral syndrome. Disease duration was calculated from the first clinical symptom to the MRI visit date.

Longitudinal data were available from four patients with additional attacks between two MRI visits, performed within one year. Phenotypes were described in detail to evaluate changes in the SC GM and WM percentages and pain in subjects with recent relapses, outside the acute stage. For this study, the “baseline” visit of each patient refers to the date when the first SC MRI was available.

### 2.2 Standard protocol approvals, registrations, and patient consent

The study was approved by the local ethics committee (EA1/131/09) and was conducted in accordance with the Declaration of Helsinki in its currently applicable version and specific German laws. All participants provided written informed consent.

### 2.3 Pain and disability assessment

A central characteristic of pain is that pain is identified from the first-person perspective. This is reflected in the current definition of pain by the International Association for the Study of Pain (IASP) as a sensory and emotional experience, giving epistemic authority to the person who qualifies their experience as pain.^1^

The Brief Pain Inventory (BPI) and the painDETECT Questionnaire (PDQ) were used to assess pain in general (BPI) and neuropathic pain (PDQ) (19–21). The BPI assesses the presence of pain, other than everyday kinds of pain such as minor headaches. It does not provide information on the type or origin of pain. It rates the average pain intensity experienced in the last 24 h as a mean of four subscales with an assessment of worst, least, average, and current by using numeric scales from 0 (“no pain”) to 10 (“pain as bad as you can imagine”). Furthermore, the BPI assesses seven domains of pain-related interference with daily life, including general activity, mood, walking ability, working ability, relations with other people, sleep, and enjoyment of life by using numeric scales from 0 to 10. A pain interference score is built as a mean of the seven subscales. Higher scores indicate worse health. The BPI reflects the current burden of pain on a person’s life.

The PDQ is designed to differentiate between nociceptive and neuropathic pain. PDQ scores ranging from 0 to 12 indicate nociceptive pain, scores from 13 to 18 indicate possible neuropathic pain, and scores from 19 to 35 indicate definite neuropathic pain. The PDQ allows for rating of average pain intensity in the last 4 weeks prior to the clinical visit by using a single numeric scale of 0–10.

Clinical disability was evaluated using the expanded disability status scale (EDSS) (22, 23), and we included sensory and pyramidal functional system scores as separate indicators for possible SC-related disability. The timed 25-foot walk (T25FW) and 9-hole peg tests (9HPT) performance scores (22, 24) were calculated by averaging the results from each repeat run. For T25FW, both forward and return runs were averaged, while for 9HPT, all runs—two per hand—were summed together and averaged.

### 2.4 MRI acquisition

Magnetic resonance imaging scans were performed on two 3-Tesla (Siemens MAGNETOM Trio Tim and Prisma, Erlangen, Germany) scanner models. The Trio MRI (Scanner 1) protocol included (1) a T1-weighted 3D magnetization prepared rapid gradient echo (MPRAGE) cerebral MRI [1 mm isotropic resolution, repetition time (TR) = 1,900 ms, time to echo (TE) = 3.03 ms], including the upper cervical cord; (2) a 2D-sagittal T2-weighted SC sequence (slice thickness = 2 mm, TR = 3,500 ms, TE = 101 ms, in-plane resolution = 0.91 mm × 0.91 mm) at the cervicothoracic and lumbar levels of the SC; and (3) a 2D phase-sensitive inversion recovery (PSIR) sequence (slice thickness = 5 mm, TR = 930 ms, TE = 3.22 ms, TI = 400 ms, in-plane resolution = 0.78mm × 0.78 mm) at the cervical C2-C3 intervertebral space level. The Prisma MRI (Scanner 2) protocol included (1) a T1-weighted 3D MPRAGE cerebral MRI (0.8 mm isotropic resolution, TR = 2,500 ms, TE = 2.22 ms, TI = 1,000 ms), including the upper cervical cord; (2) a 2D-sagittal short T1 inversion recovery (STIR) SC sequence (slice thickness = 3 mm, TR = 3,700 ms, TE = 36 ms, TI = 220 ms, in-plane resolution = 0.81 mm × 0.81 mm) at the cervicothoracic and lumbar levels; and (3) a 2D PSIR sequence with same parameters as with the Trio. No patients were imaged in the acute disease/myelitis phase or were treated with steroids less than 2 months prior to MRI scanning.

### 2.5 Spinal cord lesion analysis

SC T2-hyperintense lesion location and length (measured as vertebral segments spanned, where half segments were rounded up to the next full length) were analyzed using SC MRIs as detailed in Chien et al. (7), using 2D T2-weighted or 2D STIR SC scans. All images were reviewed and evaluated by CC (who had 8 years of experience in SC MRI reading and research). Of note, each level of cord lesion was only counted once.

### 2.6 Spinal cord gray matter and white matter segmentation

**Figure 1:**
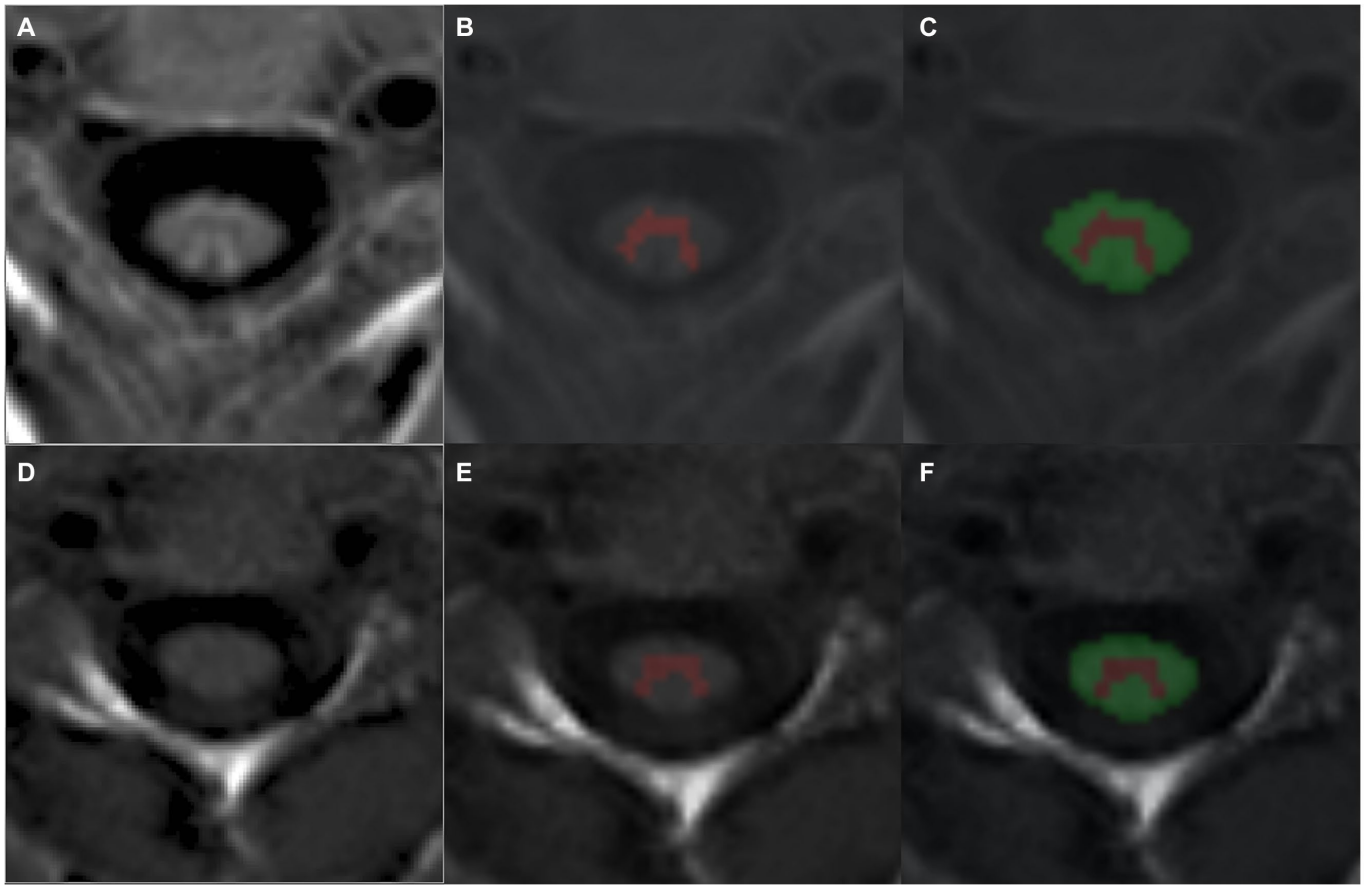
Representative segmentations of PSIR phase scans from an **(A–C)** AQP4-IgG + NMOSD and **(D–F)** a MOGAD patient using a trained CNN to automatically detect cervical cord GM **(B,E)** and WM **(C,F)**. AQP4-IgG + NMOSD, Aquaporin-4 antibody seropositive neuromyelitis optica spectrum disorders; WM, White matter; GM, Gray matter; MOGAD, Myelin oligodendrocyte associated disease.

The PSIR sequence has been previously shown to allow for robust segmentation of SC GM using both semi-and fully automated algorithms (25). Thus, for the evaluation of regional SC measures, PSIR sequences at the C2/C3 intervertebral level were collected. Fifty PSIR scans from AQP4-IgG + NMOSD, MOGAD, multiple sclerosis patients, and healthy participants were converted to NIFTI format, and WM and GM were manually segmented in the phase-sensitive image, with reference to the magnitude image when lesions in the cord occurred in the image slice using ITK-SNAP^2^ (26). These WM and GM binary masks were used to train (*n* = 35 for training, *n* = 15 for validation) a deep learning, convolutional neural network (CNN) algorithm to automatically segment the phase-sensitive images of the PSIR scans with default training hyperparameters (27) to create a final model for use in our cohort PSIR scans. Segmented WM and GM and cervical cord (WM + GM) masks were then extracted for their volumes using fslstats,^3^ and WM and GM volumes were converted to percentages by dividing by the cervical cord volume and multiplying by 100. In this study, “cervical cord volume” is defined as the combined WM and GM volume extracted from the PSIR scans. As mentioned above, four patients from the AQP4-IgG + NMOSD sub-group were removed from the cohort due to MRI quality issues (motion artifacts and/or poor image quality causing erroneous segmentation). Figure 1 shows representative automatic segmentations of PSIR scans from AQP4-IgG + NMOSD (Figures 1A–C) and MOGAD (Figures 1D–F) patients.

### 2.7 Mean upper cervical cord area analysis

All mean upper cervical cord area (MUCCA) (28) measurements were performed on 3D cerebral MPRAGE scans and blinded to patient antibody serostatus, as detailed in Chien et al. (29). Briefly, five consecutive slices, using the C2-C3 intervertebral space as a middle landmark, were segmented using JIM software (Version 7.0, http://www.xinapse.com/) and then averaged to obtain one mean cross-sectional area for each subject using an in-house python script. MUCCA was calculated in this cohort as a representative (gold-standard) total SC atrophy measure, as shown previously to be robust in AQP4-IgG + NMOSD patients (15).

### 2.8 Statistical analysis

Demographic values were compared between AQP4-IgG + NMOSD and MOGAD patients using appropriate parametric and non-parametric statistical tests as described in Table 1. Chi-squared tests were used to compare SC lesion load between patient groups. Multivariable linear regression models were applied to test associations of SC WM% and cervical cord volumes with clinical disability and pain measures, controlling for age, sex, and disease duration in each group. We chose to only perform all multivariable linear regression analyses using only the cervical cord volume and SC WM% metrics, since the SC GM% is relative to the WM%.

**Table 1:** Demographics of the study cohort.

	AQP4-IgG+ NMOSD (*n* = 20)	MOGAD (*n* = 16)	Test statistics
Age (years), Mean ± SD	49.5 ± 14.4	45.3 ± 16.9	χ^2^ = 36, *p* = 0.33
F:M, *n* (%)	19:1 (95:5)	11:5 (69:31)	χ^2^ = 4.41, ***p* = 0.04**
Disease duration (years), mean (range)	9.12 (1–28.9)	9.97 (0.2–42.6)	*t* = −0.26, *p* = 0.79
Number of subjects with a history of clinical myelitis, *n* (%)	17 (85%)	8 (50%)	χ^2^ = 5.13, *p* = 0.023^b^
Number of myelitis attacks, median (range)	1 (0–4)	0.5 (0–3)	χ^2^ = 6.51, *p* = 0.16
Time since last attack (years), mean (range)	6.56 (1–16.61)	5.08 (0.15–39.6)	*t* = −0.6, *p* = 0.56
Time since last myelitis attack (years), mean (range)	7.41 (1–15.98)	5.09 (1.46–12.77)	*t* = 0.814, *p* = 0.43
MUCCA (mm^2^), mean ± SD	68.54 ± 6.81	71.52 ± 9.69	*t* = −1.04, *p* = 0.307
Cervical cord volume (mL), mean ± SD	0.41 ± 0.04	0.44 ± 0.05	*t* = −1.83, *p* = 0.076
Cord WM percent (%), mean ± SD	80.70 ± 2.21	81.04 ± 1.77	*t* = −0.50, *p* = 0.618
Cord GM percent (%), mean ± SD	19.19 ± 2.17	18.88 ± 1.76	*t* = 0.47, *p* = 0.644
EDSS, median (range)	3.5 (0–6.5)	2.5 (0–4)	***t* = 2.12, *p* = 0.04**
25-foot walk speed (m/s), mean ± SD	1.65 ± 1.06	1.15 ± 0.2	*t* = 2.03, *p* = 0.056
9HPT performance dominant hand (s), mean ± SD	20.97 ± 3.98	22.09 ± 3.73	*t* = −0.86, *p* = 0.4
9HPT performance non-dominant hand (s), mean ± SD	42.92 ± 10.73	44.35 ± 5.09	*t* = −5.3, *p* = 0.63
Number of patients with pain (BPI), *n* (%)	14 (65%)	10 (62.5%)	χ^2^ = 0.23, *p* = 0.64
Average pain intensity within 24 h in pain patients (BPI), mean (range)	2.07 (0–6.25)	1.45 (0–5.25)	*t* = 0.88, *p* = 0.38
Number of patients with neuropathic pain, *n* (%)	8 (40%)	2 (12.5%)	n.a.
Average neuropathic pain intensity within 4 weeks (in neuropathic pain patients), mean (range)	5.5 (0–8)	4.33 (0–7)	n.a.

AQP4-IgG + NMOSD, Aquaporin-4 antibody seropositive neuromyelitis optica spectrum disorders; WM, White matter; GM, Gray matter; EDSS, Expanded disability status scale; MOGAD, Myelin oligodendrocyte associated disease; *n*, number; 9HPT, 9-Hole peg test; χ^2^, Chi-squared; and *t*, *t*-statistic. Bold text indicates statistical significance.

Cervical cord volume extracted from PSIR scans was compared with MUCCA (gold standard) using a Pearson’s correlation test as an evaluation for the robustness of the automated SC segmentation in this cohort. Pearson’s correlation test was used to analyze the association between GM and WM volumes, as well as GM% and WM% with cervical cord volume in both patient groups.

Comparison within and between group SC WM%, GM%, and cervical cord volume were performed using Welch two-sample *t*-tests and effect sizes were evaluated using Cohen’s *d*, which were also applied to compare these SC metrics in patients with and without pain.

Multivariable linear regression modeling in each patient group to investigate associations between time since the last clinical attack with SC WM% and cervical cord volumes included age and sex as covariates.

In all tests, a value of *p* of <0.05 was considered statistically significant. All data were analyzed and graphs were created using R.^4^ The longitudinal case series data were presented without statistical analysis due to the small sample size (*n* = 4).

## 3 Results

### 3.1 Cohort demographics

Demographics of AQP4-IgG + NMOSD and MOGAD patients in this cohort are shown in Table 1.

Fourteen subjects with AQP4-IgG + NMOSD and 10 subjects with MOGAD experienced pain, other than everyday kinds of minor pain at the time of assessment. Eight subjects with AQP4-IgG + NMOSD and two subjects with MOGAD fulfilled the criteria for neuropathic pain. Pain courses comprised persistent pain with slight fluctuations (*n* = 3), persistent pain with pain attacks (*n* = 4), and pain attacks without pain between them (*n* = 3). The pain was located on one or several parts of the body, including legs (*n* = 10), trunk (*n* = 9), feet (*n* = 4), hip/bottom (*n* = 3), hands (*n* = 2), arms (*n* = 1), and head (*n* = 1). The pain was described to be electric-like (*n* = 9), tingling (*n* = 9), burning (*n* = 6), associated with mechanical (*n* = 8) and thermal (*n* = 8) allodynia, and/or in an area of numbness (*n* = 9).

Table 2 provides a summary of the SC lesion count and spinal cord levels affected in each patient group.

**Table 2:** Patients with T2-hyperintense spinal cord lesions counts and levels of injury.

	AQP4-IgG + NMOSD (*n* = 20)	MOGAD (*n* = 16)	Test statistics
Spinal cord lesions, *n* (%)	10 (50%)	6 (37.5%)	χ^2^ = 0.56, *p* = 0.45
Cervical cord lesions (Dens–C7), *n* (%)	6 (30%)	4 (25%)	χ^2^ = 0.11, *p* = 0.74
Cervico-thoracic lesions, *n* (%)	2 (10%)	1 (6.25%)	χ^2^ = 0.16, *p* = 0.69
Thoracic cord lesions, *n* (%)	2 (10%)	1 (6.25%)	χ^2^ = 0.16, *p* = 0.69
History of myelitis, *n* (%)	17 (85%)	8 (50%)	**χ**^**2**^ **= 5.13, *p* = 0.02**

AQP4-IgG+, Aquaporin-4 antibody seropositive; NMOSD, Neuromyelitis optica spectrum disorders; MOG-IgG+, Myelin oligodendrocyte glycoprotein antibody; χ^2^, Chi-square; *n*, Number. Bold text indicates statistical significance.

### 3.2 Cervical cord volume and WM percentage associations with pain and disability measurements

In AQP4-IgG + NMOSD, the cervical cord volume was associated with mean pain severity within 24 h. The lower the cervical cord volume, the higher the pain intensity (Table 3).

**Table 3:** SC cervical cord volume and WM percentage associations with pain severity and interference within the previous 24 h of assessment.

Cervical cord volume		
	AQP4-IgG + NMOSD	MOGAD
Mean pain severity within 24 h	**ß = −0.62, *p* = 0.009**	ß = 0.12, *p* = 0.707
Mean pain interference within 24 h	**ß = −0.56, *p* = 0.010**	ß = 0.04, *p* = 0.896
SC WM percentage		
	AQP4-IgG + NMOSD	MOGAD
Mean pain severity within 24 h	ß = −0.26, *p* = 0.323	ß = 0, *p* = 0.996
Mean pain interference within 24 h	ß = −0.34, *p* = 0.157	ß = −0.05, *p* = 0.871

AQP4-IgG + NMOSD, Aquaporin-4 antibody seropositive neuromyelitis optica spectrum disorders; MOGAD, Myelin oligodendrocyte antibody associated disease; and WM, White matter. Bold text indicates statistical significance (*p* < 0.05).

Neither WM% or GM% differed in AQP4-IgG + NMOSD patients with and without pain (WM%: *t* = −0.18, *p* = 0.862, Cohen’s *d* = −0.09, GM%: *t* = 0.16, *p* = 0.873, Cohen’s *d* = 0.08). In MOGAD, both cervical cord volume and WM% did not differ between patients with and without pain (cervical cord volume: *t* = −0.55, *p* = 0.605, Cohen’s *d* = −0.37; WM%: *t* = −0.42, *p* = 0.680, Cohen’s *d* = −0.21; GM%: *t* = 0.43, *p* = 0.677, Cohen’s *d* = 0.21). There was a difference in cervical cord volumes between AQP4-IgG + NMOSD patients with pain vs. those without (*t* = −2.3, *p* = 0.042, Cohen’s *d* = −1.2), where patients with pain had on average lower cervical cord volumes than those without pain.

**Figure 2:**
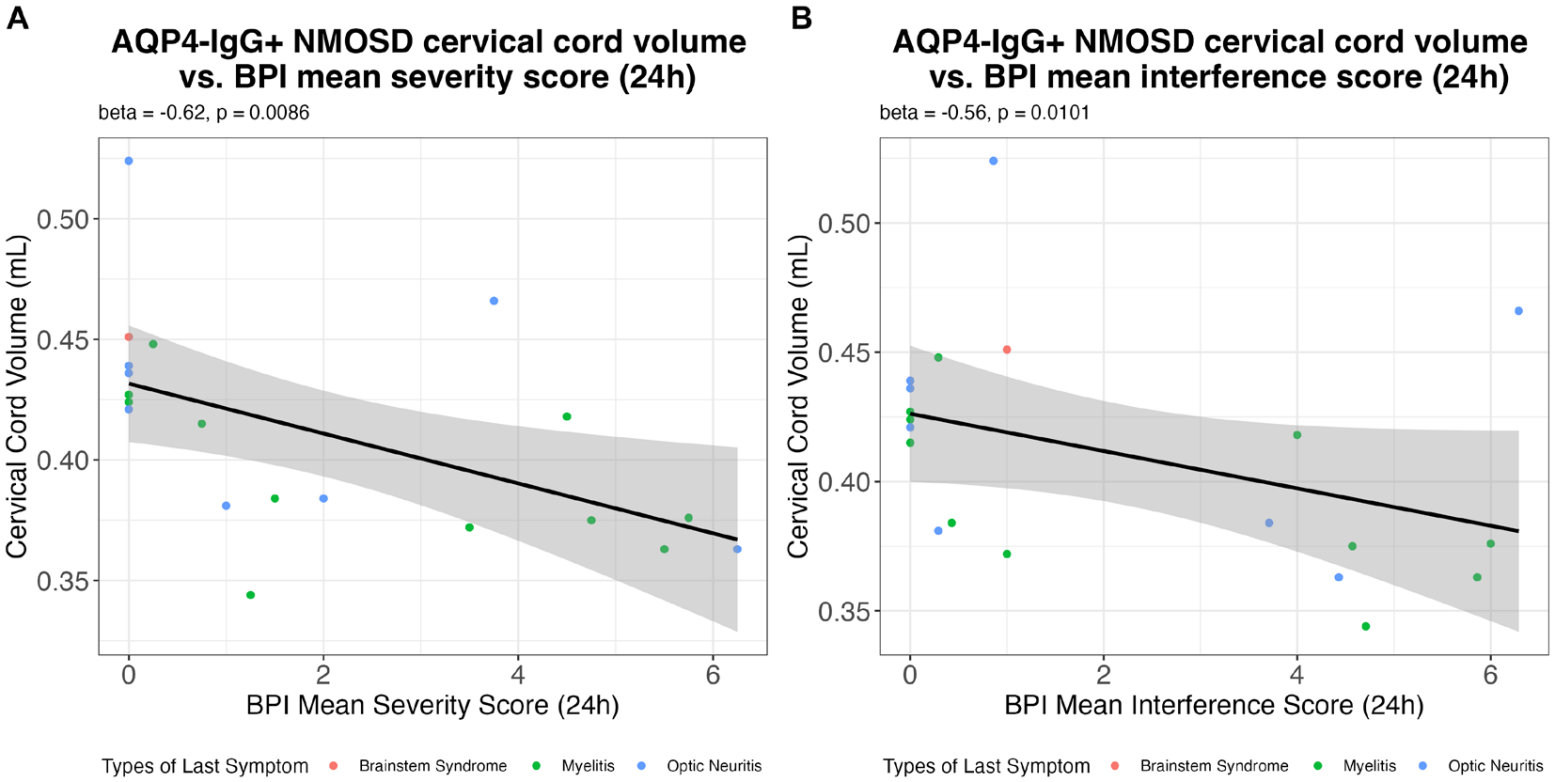
The association of the **(A)** BPI mean pain severity score; and **(B)** BPI mean interference score in the previous 24 h with cervical cord volume (mL) in AQP4-IgG + NMOSD. AQP4-IgG + NMOSD, Aquaporin-4 antibody seropositive neuromyelitis optica spectrum disorders; BPI, Brief pain inventory.

Figure 2 illustrates the associations found between BPI pain severity and intensity vs. cervical cord volume in AQP4-IgG + NMOSD patients.

No significant associations were found between cervical cord volume and SC WM percentage with pain metrics, EDSS, or the sensory and pyramidal functional systems scores (Table 4).

**Table 4:** Cervical cord volume and SC WM percentage associations with clinical disability.

Cervical cord volume		
	AQP4-IgG + NMOSD	MOGAD
EDSS	β = 0.14, *p* = 0.516	β = −0.31, *p* = 0.242
Sensory EDSS	β = −0.26, *p* = 0.307	β = −0.41, *p* = 0.149
Pyramidal EDSS	β = −0.24, *p* = 0.166	β = −0.30, *p* = 0.296
SC WM percentage		
	AQP4-IgG + NMOSD	MOGAD
EDSS	β = −0.07, *p* = 0.762	β = −0.36, *p* = 0.182
Sensory EDSS	β = −0.01, *p* = 0.973	β = −0.45, *p* = 0.112
Pyramidal EDSS	β = −0.23, *p* = 0.191	β = −0.21, *p* = 0.473

AQP4-IgG + NMOSD, Aquaporin-4 antibody seropositive neuromyelitis optica spectrum disorders; EDSS, Expanded disability status scale; and MOGAD, Myelin oligodendrocyte associated disease.

No associations were found between cervical cord volume or SC WM% and 9HPT test times or T25FW speed (shown in Supplementary Table 1).

### 3.3 Cervical cord volume in relation to MUCCA and WM, GM volumes, and percentages

To check the segmentation of SC regions using the CNN algorithm, we tested the cervical cord volume against MUCCA, the gold standard in SC atrophy measures (28), in the whole cohort. To evaluate the contributions of GM and WM volumes and percentages from each group at the C2/C3 level to the cervical cord volume, we similarly conducted Pearson’s correction tests (Supplementary Figure 1).

The cervical cord volume and MUCCA were strongly associated (*R* = 0.51, *p* = 0.0015). Interestingly, the cord GM volume was highly associated with cervical cord volume in MOGAD, while there was slightly less contribution of the GM volume to the PSIR cervical cord volume in AQP4-IgG + NMOSD patients (*R* = 0.69, *p* = 0.0033 and *R* = 0.53, *p* = 0.015, respectively; Supplementary Figure 1B). Cord WM volume was highly associated with cervical cord volume in both cohorts (AQP4-IgG + NMOSD: *R* = 0.98, *p* = 2e−13 and MOGAD: *R* = 0.99, *p* = 3.9e−12; Supplementary Figure 1C). Our analysis revealed similar non-significant trends with AQP4-IgG + NMOSD and MOGAD GM% decreasing, with larger cervical cord volumes. The opposite trend was also seen in the WM% vs. cervical cord volume in both disease groups (Supplementary Figures 1D,E).

### 3.4 Cervical cord volume, GM, and WM percentage comparisons between patient groups

**Figure 3:**
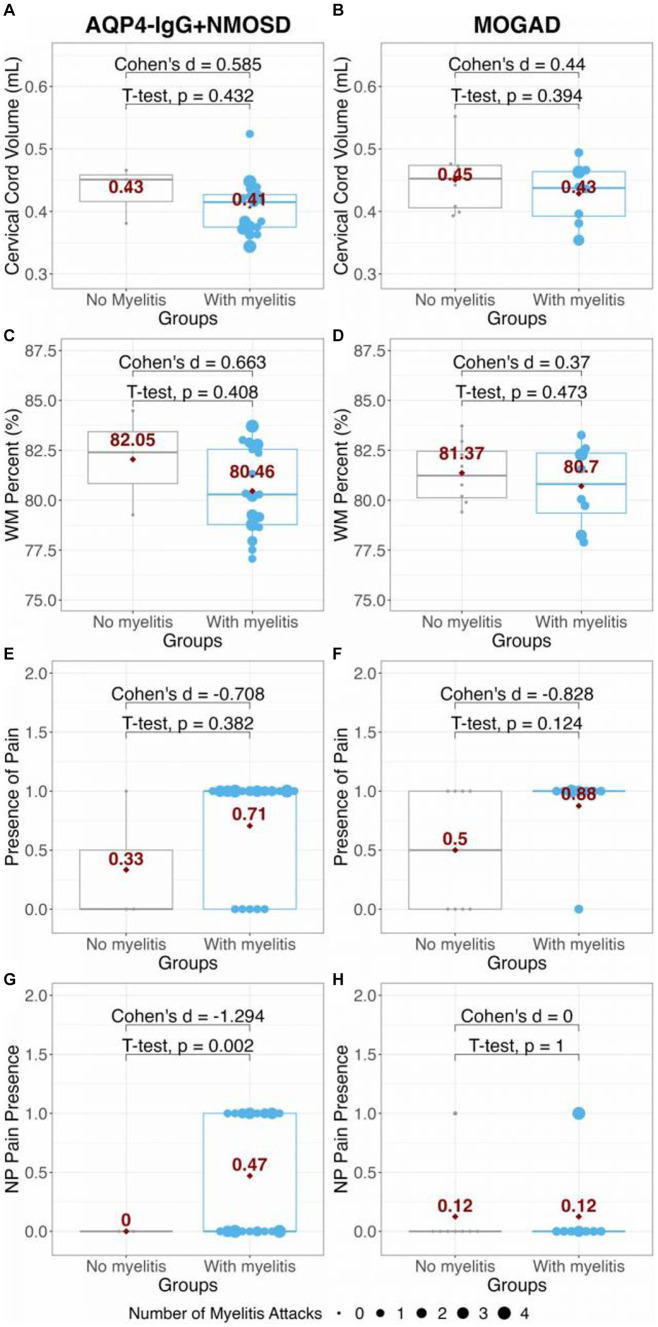
Comparison of panels **(A,B)** cervical cord volume; and **(C,D)** WM percent in AQP4-IgG + NMOSD and MOGAD patients. The presence of pain and presence of NP in AQP4-IgG + NMOSD and MOGAD patients are shown in panels **(E–H)**. The red dots and numbers indicate the mean values of each metric per group. AQP4-IgG + NMOSD, Aquaporin-4 antibody seropositive neuromyelitis optica spectrum disorders; MOGAD, Myelin oligodendrocyte associated disease; WM, White matter; and NP, Neuropathic pain.

No significant differences in cervical cord volume or WM% were found between AQP4-IgG + NMOSD or MOGAD at the C2/C3 level of the cord (*t* = −1.84, *p* = 0.076, Cohen’s *d* = −0.626; *t* = −0.503, *p* = 0.618, Cohen’s *d* = −0.165; respectively). There was also no difference found between group SC GM% (*t* = 0.47, *p* = 0.644, Cohen’s *d* = −0.621). However, it was observed that both patient groups with a history of myelitis attacks showed a general (but not significant) decrease in both cervical cord volume and SC WM percentage compared to patients without myelitis attacks within each group (Figure 3). As a consequence of GM% being complementary to the WM%, in both groups, there was a slight, non-significant increase in SC GM% in patients with a history of myelitis attacks (data not shown). From Figure 3, it can be seen that 71% (12 of 17) of AQP4-IgG + NMOSD and 88% (7 of 8) of MOGAD patients with a history of myelitis attacks reported current pain. Meanwhile, 47% (8 of 17) of AQP4-IgG + NMOSD and only 13% (1 of 8) of MOGAD patients with previous myelitis reported neuropathic pain. Thus, we did not test associations of neuropathic pain with SC measures further in the MOGAD patients.

### 3.5 Time since last attack, SC cervical volume, and WM percentage

No associations were found when evaluating cervical cord volume (AQP4-IgG + NMOSD: β = 0.20, *p* = 0.419; MOGAD: β = −0.23, *p* = 0.438), and time passed since the last clinical symptom of any type. In AQP4-IgG + NMOSD, we observed some SC WM% decreased over time after an attack (β = −0.41, *p* = 0.096). In MOGAD, this trend was not observed (β = −0.16, *p* = 0.570).

### 3.6 Longitudinal case series of patients with additional attacks between follow-up visits

Out of the 35 patients included in this study, four patients with longitudinal clinical and MRI data were found to have an additional attack between visits. Supplementary Table 2 shows an in-depth qualitative assessment of these patients in relation to their attack history, prevalence of pain, and SC MRI findings.

Subject 1 (AQP4-IgG + NMOSD) experienced an optic neuritis attack 1 month prior to the second SC scan (two optic neuritis attacks in total between visits). This patient showed an increased GM% in the cord of approximately 2% (from 0.079 to 0.091 mL) at follow-up, with an increase in cervical cord volume (from 0.38 to 0.40 mL) and relatively stable WM volume (0.302–0.308 mL). Interestingly, although pain intensity was stable in this patient, the pain location expanded from the feet to the legs and additionally to the head/neck during the second visit.

Subject 2 was also diagnosed with AQP4-IgG + NMOSD and experienced a myelitis attack 5 months prior to the follow-up visit. Again, GM% was increased by roughly 3% (from 0.070 to 0.079 mL), but with an associated 3% decrease in WM% (from 0.347 to 0.317 mL) and a decrease in cervical cord volume (from 0.42 to 0.40 mL). In this patient, the neuropathic pain score increased from baseline to follow-up.

Two MOGAD patients (Subjects 3 and 4) had a visit more than 6 months after a myelitis attack. Both subjects showed a GM% decrease of ~1–2% between the two visits, along with a decrease in the cervical cord volume. In both MOGAD patients, pain decreased over time.

## 4 Discussion

Spinal cord affection is a central issue in AQP4-IgG + NMOSD and MOGAD. Our study highlights regional SC affection in these diseases and its relation to pain intensity:

1. Higher mean pain severity and pain interference with daily-life activities in AQP4-IgG + NMOSD patients were significantly associated with decreased cervical cord volume at the C2-C3 cord level.
2. GM volume at the C2-C3 intervertebral level tended to contribute more to cervical cord volume in MOGAD than in AQP4-IgG + NMOSD patients.
3. WM percentage in AQP4-IgG + NMOSD patients tended to decrease after a myelitis attack (non-significant).
4. Two AQP4-IgG + NMOSD patients with clinical attacks between longitudinal follow-up visits showed an increase in GM% (2–3%), while two MOGAD patients showed a decrease in GM% (1–2%).

### 4.1 Association of pain intensity and cervical cord volume

Pain is one of the most disabling symptoms of AQP4-IgG + NMOSD patients and has a severe impact on the quality of life (13). Pain syndromes have various origins, comprising among others myelitis-mediated neuropathic pain, ON-related headaches, musculoskeletal pain, and spasticity-related pain (13). The origin of pain is often complex and difficult to determine and it is important to note that pain is always an individual experience, comprising sensory and emotional aspects. Unfortunately, structural pain assessment is frequently lacking in clinical practice and the investigation of associations between pain and structural disease markers is scarce. In our cohort, the examination of the interrelation between cervical cord volume on the cervical level and measurements of pain revealed a negative association with the mean pain severity and pain interference with daily-life activities in AQP4-IgG + NMOSD patients. These findings add to our previous study, showing an inverse correlation of the thalamic VPN volume with pain intensity in AQP4-IgG + NMOSD (30). An association between lower cervical cord volume and higher pain intensity through both damage of decussating fibers of the spinothalamic tract as previously described in MS (31) and through myelitis-related GM damage is conceivable (32, 33). Further exploration is needed to detect if attack-independent tissue damage (34) may contribute to SC atrophy (16, 17), and thus aggravate pain over time. Moreover, longitudinal pain assessment from disease onset is necessary to analyze pathogenic interrelations between the origin of pain and structural pathology. Consequently, pain assessment is necessary to rate disease-related disability beyond the EDSS (35) and to gain further insights into the disease mechanisms of AQP4-IgG + NMOSD.

No associations occurred between WM percentages and clinical pain measurements in AQP4-IgG + NMOSD patients and between SC cervical volume or WM percentage and clinical pain measurements in MOGAD patients. Our findings are consistent with the literature showing that MOGAD has a lower clinical myelitis incidence with a smaller chronic lesion burden (7) and a lower impact of chronic pain syndromes than AQP4-IgG + NMOSD (14). Larger study samples are needed to detect a potential association between pain and spinal cord affection in MOGAD.

In line with previous studies, overall clinical disability, as measured by EDSS, was higher in AQP4-IgG + NMOSD than in MOGAD patients (7, 9). However, this difference in general disability was not reflected in cross-sectional cervical cord volume and/or WM percentage in the cervical cord measured using PSIR scans. Furthermore, cervical cord volume and SC WM percentages were not associated with the EDSS, 9HPT, and T25FW in either patient group. Although our AQP4-IgG + NMOSD cohort had a higher EDSS at baseline compared to that of the MOGAD cohort, the effect of SC injury may not be well reflected in EDSS scores ≤4 (36).

### 4.2 Cervical GM and WM profiles in AQP4-IgG + NMOSD and MOGAD

MUCCA has previously been shown to reflect total cord volumes in AQP4-IgG + NMOSD (28). Our findings that the cervical cord volumes are highly correlated with MUCCA in the entire cohort indicate that our method is reliable for evaluation of SC changes in these diseases. When investigating WM and GM regional contributions to the cervical cord volume, we found that in both diseases, GM volume increased with increasing cervical cord volume. However, the AQP4-IgG + NMOSD patient cervical cord volumes were generally lower and this correlation analysis showed less contribution of the GM volume to the cervical cord volume than in MOGAD. The range of SC GM% measured in our entire cohort is similar to the 15–20% GM proportion found in a post-mortem study in healthy donor spines (37). Interestingly, the mean SC GM% of AQP4-IgG+ patients was higher than in MOGAD, although not statistically significant. Assuming that both diseases are associated with a reduced SC volume, these findings could indicate that WM pathology contributes to pain increase in NMOSD. Due to the small sample size, this theory remains speculative and should be investigated in a larger cohort.

### 4.3 Associations of SC metrics with clinical attacks

Generally, we observed that AQP4-IgG + NMOSD patients had lower cervical cord volumes than MOGAD, both without and with myelitis. When assessing how the last clinical attack(s) influences the SC, we observed that AQP4-IgG + NMOSD patients tended toward cord WM% decrease after any type of disease-related attack. These observations were cross-sectional and did not include MRIs close to an attack. Our observation could indicate continued SC WM degeneration after AQP4-IgG + NMOSD-related attacks (33), although this decrease in WM could be due to an increase in the GM% when the primary lesional site is located purely in the GM (38). SC GM has also been found to be about twice as stiff as SC WM, such that mechanical compression of the WM is much easier, especially in the axial plane (to which our PSIR sequence was oriented) (39). It stands to reason that GM inflammation, edema, or astrogliosis could cause this region to expand and effectively compress the WM in the SC. Thus, it may well be important to evaluate both GM and WM in relation to time since an acute attack for dynamic changes in the SC.

We did not restrict our analysis to only myelitis attacks as it is unclear how non-attack-related regions are affected by either disease. In one study, it has been found that SC and retinal measures are associated with multiple sclerosis, suggesting a whole CNS pathology may occur during the disease course (40). Furthermore, we have previously shown that combinations of attacks are associated with damage outside of the attack location (34). Thus, we included PSIR SC analysis in this study from patients who did not only have a myelitis attack, although myelitis does increase SC atrophy in these patients (7), which was also evident in our current findings.

### 4.4 Longitudinal case series

In one AQP4-IgG + NMOSD patient, SC GM% increase was evident after an optic neuritis attack with stable WM% and cervical cord volume, as well as stable pain intensity.

Meanwhile, in another AQP4-IgG + NMOSD patient, there was an emergence of neuropathic pain after a myelitis attack five months prior to the follow-up visit. This characteristic is conceivably linked to the SC GM% increase with WM% decrease and myelitis attack.

Since AQP4 is expressed on astrocytes, and AQP4-IgG has been shown to cause a complement-mediated injury of astrocytes and neurons, this injury could lead to astrogliosis and cellular hypertrophy in the GM (41). As a consequence, secondary WM damage could occur nearby (42). Thus, our observation of increased GM% in the SC in two AQP4-IgG + NMOSD patients indicates that systemic inflammatory mechanisms (43) may be at play in this disease, even when an attack occurs elsewhere in the CNS, e.g., in the optic nerves.

In both MOGAD patients, cervical cord volume and GM% were decreased with additional myelitis attacks, however pain was decreased. These observations are in line with a previous study, where GM volume was found to be decreased in MOGAD patients who had resolved SC lesions compared to MOGAD patients without any lesions/myelitis attacks (9).

### 4.5 Limitations

The main limitation of our study is that our sample sizes are generally small and differ between AQP4-IgG + NMOSD and MOGAD patients. This limitation did not allow us to evaluate our data with true statistical equivalency and might have concealed significant findings in the trends we observed. Furthermore, the sample size restricted us from performing an extensive sub-analysis of subjects with myelitis as well as with respect to different pain syndromes. Future studies will benefit from including detailed pain information beyond questionnaire-based assessment. Still, we found a negative association between pain intensity and SC cervical cord volume in AQP4-IgG + NMOSD. Of note, our data do not give any information about the origin of pain and the pain duration. We therefore cannot show a causal relationship between the origin of pain and the SC volume.

Another limitation is the cross-sectional and non-acute design of this study, which did not allow for measurements of SC WM/GM changes over time. Furthermore, our longitudinal case series does not allow for definite pathogenic conclusions. However, we were able to detail qualitative and quantitative phenotypes of clinical and imaging metrics from four subjects with clinical attacks within one year. Future studies with MRI scans acute to a disease-related attack, larger sample sizes, and longitudinal follow-ups are required to investigate our hypotheses further.

Furthermore, our study lacks a SC imaging dataset from healthy controls. Our findings that the cervical cord volumes extracted from our PSIR sequence are highly associated with MUCCA, the gold standard in evaluating SC atrophy in NMOSD (28), however indicate that the regional SC metrics we extracted are reliable. Moreover, it has previously been shown that MUCCA is decreased in NMOSD, but not in MOGAD compared to healthy controls (7). Similarly, our study provides indications of generally lower cervical cord volumes in AQP4-IgG + NMOSD patients with and without myelitis compared with MOGAD. Future inclusion of healthy participant PSIR-extracted WM and GM SC volumes would benefit evaluations of attack-related damage to different SC regions in these two rare diseases and the collection of this MRI sequence is warranted.

## 5 Conclusion

Our study shows that in AQP4-IgG + NMOSD, lower SC volume was associated with increased pain intensity. This finding highlights the relevance of thorough pain workup and MRI monitoring during the disease course. Clinically feasible 2D PSIR MRIs at the C2/C3 intervertebral SC level can be reliably segmented using an automated CNN algorithm, even in a longitudinal setting. Interestingly, our MRI results indicate a different affection of GM volumes relative to SC volume between the two disease entities based both on the cross-sectional data and longitudinal data. Larger studies in both diseases with longitudinal and acute monitoring to evaluate if patients show true atrophy and pain symptom evolution, even without additional myelitis attacks are required.

## Data availability statement

The raw data supporting the conclusions of this article will be made available by the authors, without undue reservation.

## Ethics statement

The studies involving humans were approved by Charité’s Ethics Committee, Charité Universitätsmedizin, Berlin, Germany. The studies were conducted in accordance with the local legislation and institutional requirements. The participants provided their written informed consent to participate in this study.

## Author contributions

SA: Conceptualization, Data curation, Investigation, Methodology, Project administration, Validation, Writing – original draft, Writing – review & editing. OZ: Writing – original draft, Writing – review & editing. LB: Formal Analysis, Writing – review & editing. BP: Formal Analysis, Writing – original draft, Writing – review & editing. PS: Data curation, Writing – review & editing. TS-H: Data curation, Writing – review & editing. FP: Conceptualization, Supervision, Writing – review & editing. CC: Conceptualization, Formal Analysis, Methodology, Project administration, Supervision, Validation, Writing – original draft, Writing – review & editing.

## Footnotes

^1^ https://www.iasp-pain.org/

^2^ www.itksnap.org

^3^ https://fsl.fmrib.ox.ac.uk/fsl/fslwiki/Fslutils

^4^ https://www.r-project.org/

## Supplementary material

SUPPLEMENTARY FIGURE 1 Association of MUCCA with **(A)** total cord volume. Associations of cervical cord volume with **(B)** cord GM volume, **(C)** cord WM volume, **(D)** cord GM percent, and **(E)** cord WM percent extracted from segmented PSIR images at the C2/C3 level. AQP4-IgG+NMOSD, Aquaporin-4 antibody seropositive neuromyelitis optica spectrum disorders; MOGAD, Myelin oligodendrocyte associated disease; MUCCA, Mean upper cervical cord area.
