# Supplementary material for: Regional spinal cord volumes and pain profiles in AQP4-IgG + NMOSD and MOGAD
Source: Front Neurol. 2024 Jan 26;15:1308498. doi: 10.3389/fneur.2024.1308498 (PMC10853470; doi:10.3389/fneur.2024.1308498)
Supplement: Supplementary file 2 [file Table_1.pdf]

Supplementary Material

Supplemental Table 1 | Associations of cervical cord volumes and SC WM percentage with the 9HPT and T25FW

**Cervical cord volume**		
	**AQP4-IgG+NMOSD**	**MOGAD**
9HPT dominant hand	ß=0.27, p=0.162	ß=-0.19, p=0.514
9HPT non-dominant hand	ß=0.13, p=0.571	ß=0.04, p=0.874
T25FW	ß=-0.21, p=0.443	ß=-0.41, p=0.096
**SC WM percentage**		
	**AQP4-IgG+NMOSD**	**MOGAD**
9HPT dominant hand	ß=0.14, p=0.487	ß=0.12, p=0.678
9HPT non-dominant hand	ß=0, p=0.999	ß=0.45, p=0.083
T25FW	ß=-0.15, p=0.579	ß=0.31, p=0.225

AQP4-IgG+NMOSD, aquaporin-4 antibody seropositive neuromyelitis optica spectrum disorders; MOGAD, myelin oligodendrocyte antibody associated disease; T25FW, timed 25-foot walk test mean time; 9HPT. 9-hole peg test mean time.

Supplemental Table 2 | Descriptive longitudinal clinical and SC phenotypic case series of patients with additional attacks between MRI visits

Patient	Clinical History	SC MRI findings
Subject 1, F, 51y<br><br>Diagnosis: AQP4-IgG+NMOSD<br><br>Age at onset: 22 y<br><br>Attacks before Baseline<br>- Over 29 years after disease onset multiple episodes of ON (1/year)<br>- 25 years after disease onset single episode of Myelitis and ON<br><br>Prior immunotherapy<br>- Single administration of mitoxantrone, discontinuation due to sepsis<br>- Octagam: 43 months<br>- Single administration of Rituximab (persistent B-cell depletion)	**Baseline visit 09/2017:**<br><br>Disease duration: 29 y<br><br>EDSS: 3.5<br><br>Pain location: feet<br><br>Average Pain Intensity 24h: 0/10<br><br>Average Pain Intensity 4w: 1/10<br><br>Neuropathic Pain Score: 7/35<br><br>No immunotherapy<br><br>**Clinical visit 10/2018:**<br><br>Attack 04/2018: ON RE<br><br>Symptoms: Visual impairment restricted walking distance and impaired balance<br><br>Therapy: IVMP<br><br>Attack 09/2018: ON RE	**09/2017: Scanner 1**<br><br>GM (mL): 0.079 (20.73%)<br><br>WM (mL): 0.302 (79.27%)<br><br>Cervical Cord (mL): 0.38<br><br>**10/2018: Scanner 1**<br><br>GM (mL): 0.091 (22.81%)*<br><br>WM (mL): 0.308 (77.19%)**<br><br>Cervical Cord (mL): 0.40

No relevant comorbidities	Symptoms: Visual impairment<br><br>Therapy: 6x IA<br><br><br>EDSS: 4.0<br><br>Pain location: Head/neck, legs<br><br>Average Pain Intensity 24h: 1/10<br><br>Neuropathic Pain Score: 6/35<br><br>No immunotherapy	*It can be seen that 1 month after an ON attack, the GM percent in the SC increased by 2% from baseline and the cervical cord volume also increased.<br><br><br>**WM% does not increase, and as it is complementary to the GM % in the cord, it decreased over time.
Subject 2, F<br><br><br>Diagnosis: AQP4-IgG+NMOSD<br><br>Age at onset: 60y<br><br><br>Attacks before baseline<br><br>- One myelitis<br><br><br>No prior immunotherapy	**Baseline Visit 11/2017:**<br><br>Disease duration: 11 months<br><br>EDSS: 6.5<br><br>Pain location: trunk<br><br>Average Pain Intensity 24h: N.A.<br><br>Average Pain Intensity 4w: 10/10<br><br>Neuropathic Pain Score: 8/35<br><br>No Immunotherapy<br><br><br>**Clinical Visit 11/2018:**<br><br>Attack 06/2018: Myelitis	**11/2017: Scanner 1**<br><br>GM (mL): 0.07 (16.75%)*<br><br>WM (mL): 0.347 (83.01%)**<br><br>Cervical Cord (mL): 0.42<br><br><br><br>**11/2018: Scanner 1**<br><br>GM (mL): 0.079 (19.95%)*

Comorbidities:<br>- Spinal canal stenosis C4-6 with intervertebral disc graft C4/5 without myelopathy<br>Recurrent myoclonus attacks of the arms without loss of consciousness (differential diagnosis: spinal myoclonus)	Symptoms: sensorimotor spinal cord syndrome below Th10, bladder and bowel disturbance<br><br>Therapy: N.A., no IVMP<br><br>EDSS: 6.0<br><br>Pain location: trunk<br>Average Pain Intensity 24h: 8/10<br>Average Pain Intensity 4w: N.A.<br>Neuropathic Pain Score: 15/35<br><br>Immunotherapy: Rituximab since 12/2017	WM (mL): 0.317 (80.05%)**<br>Cervical Cord (mL): 0.40<br>*It can be seen that with an additional myelitis within 5 months of the second MRI visit, the GM% increased by 3%, even though the cervical cord volume decreased.<br><br>**WM% also decreases (~3% between visits), and as it is complementary to the GM % in the cord.
Subject 3, F, 51 y<br><br>Diagnosis: MOGAD<br>Age at onset: 47y<br>Attacks before baseline<br>- Four myelitis attacks<br>- Three brainstem attacks	**Baseline Visit 09/2018:**<br>Disease duration: 30 months<br>EDSS: 3.5<br>Pain location: Head/neck, trunk, hip, legs, feet<br>Average Pain Intensity 24h: 5.5/10	**09/2018: Scanner 1**<br>GM (mL): 0.082 (17.71%)*<br>WM (mL): 0.381 (82.29%)**<br>Cervical Cord (mL): 0.46

Prior immunotherapy<br><br>- Three cycles of Rituximab<br>- Mycophenolate mofetil + Prednisolon since 02/2017<br><br>Comorbidities<br><br>- Cervical spine syndrome<br>- Vitamin D deficiency<br>- Migraine<br>- Anxiety disorder	Average Pain Intensity 4w: 5/10<br><br>Neuropathic Pain Score: 17/35<br><br>Immunotherapy: Mycophenolate mofetil since 02/2017.<br><br>**Clinical Visit 09/2020:**<br><br>Attack 01/2019: Myelitis<br><br>Symptoms: Paresthesia, Hyperalgesia<br><br>Therapy: IVMP, PLEX<br><br>Pain location: Head/neck, trunk, hip, legs, feet<br><br>Average Pain Intensity 24h: 4.75/10<br><br>Average Pain Intensity 4w: 5/10<br><br>Neuropathic Pain Score: 22/35<br><br>Immunotherapy: Tocilizumab + IVIG since 04/2020	**09/2020: Scanner 2**<br><br>GM (mL): 0.067 (16.30%)*<br><br>WM (mL): 0.344 (83.70%)**<br><br>Cervical Cord (mL): 0.41

	**Clinical Visit 08/2021:**<br><br>Attack 12/2020: Myelitis<br><br>Symptoms: Numbness in the area of the anus - aggravation of previous symptoms (burning whole body pain, backpain)<br><br>Therapy: oral prednisone, PLEX<br><br>Pain location: Head/neck, trunk, hip, legs, feet<br><br>Average Pain Intensity 24h: 2/10<br><br>Average Pain Intensity 4w: 3/10<br><br>Neuropathic Pain Score: 22/35<br><br>Immunotherapy: Tocilizumab + IVIG since 04/2020	**08/2021: Scanner 2**<br><br>GM (mL): 0.061 (15.17%)*<br><br>WM (mL): 0.341 (84.83%)**<br><br>Cervical Cord (mL): 0.40<br><br>*It can be seen that the GM% consistently decreases by ~1% between visits and the cervical cord volume also decreases with additional myelitis attacks between visits.<br><br>**WM% also increases (~1% per visit), and as it is complementary to the GM % in the cord.

Subject 4, F<br><br>Diagnosis: MOGAD<br><br>Age at onset: 53 y<br><br>Attacks before baseline<br>- Two confirmed brainstem attacks, one including the cervical myelon	**Baseline Visit 09/2018:**<br><br>Disease duration: 29 months<br><br>EDSS: 2.0<br><br>Average Pain Intensity 24h: 5/10<br><br>Average Pain Intensity 4w: 7/10<br><br>Neuropathic Pain Score: 8/35<br><br>No Immunotherapy	**09/2018: Scanner 1**<br><br>GM (mL): 0.073 (18.43%)*<br><br>WM (mL): 0.323 (81.57%)**<br><br>Cervical Cord (mL): 0.40
Prior immunotherapy<br>- Azathioprine: 3 months (discontinuation due to elevation of liver transaminases)<br>- Low-dose prednisone p.o.: 24 months<br><br>No relevant comorbidities	**Clinical Visit 10/2019:**<br><br>Attack 04/2019: Myelitis<br><br>Symptoms: Sensory spinal cord syndrome with dysesthesia below Th12<br><br>Therapy: IVMP<br><br>EDSS 3.0<br><br>Pain location: Head/neck, arms, hands, legs, feet<br><br>Average Pain Intensity 24h: 6/10<br><br>Average Pain Intensity 4w: 0/10<br><br>Neuropathic Pain Score: 6/35	**10/2019: Scanner 1**<br><br>GM (mL): 0.061 (16.27%)*<br><br>WM (mL): 0.314 (83.73%)**<br><br>Cervical Cord (mL): 0.38<br><br>*It can be seen that with an additional myelitis attack between visits, the GM% was decreased by ~2% in a year and the cervical cord volume decreased as well.

	Immunotherapy: Rituximab since 03/2019	**WM% increases, and as it is complementary to the GM % in the cord.

AQP4-IgG+NMOSD, Aquaporin-4-IgG positive neuromyelitis optica spectrum disorder; EDSS, Expanded disability status scale; F, female; GM, grey matter; h, hours; IA, immunoadsorption; IVMP, intravenous methylprednisolone; m, male; mL, milliliters; MOGAD, myelin oligodendrocyte glycoprotein associated disease; PLEX, plasma exchange; p.o., per os; RE, right eye; SC, spinal cord; w, weeks; WM, white matter; y, years. Asterisks denote observational notes made in each patient visit pertaining to their SC MRI.
